# Role of Primary Afferents in Arthritis Induced Spinal Microglial Reactivity

**DOI:** 10.3389/fimmu.2021.626884

**Published:** 2021-04-07

**Authors:** Charlie H. T. Kwok, Yuta Kohro, Michael Mousseau, Melissa S. O’Brien, John R. Matyas, Jason J. McDougall, Tuan Trang

**Affiliations:** ^1^ Comparative Biology and Experimental Medicine, University of Calgary, Calgary, AB, Canada; ^2^ Physiology and Pharmacology, Hotchkiss Brain Institute, University of Calgary, Calgary, AB, Canada; ^3^ Department of Molecular and System Pharmacology, Graduate School of Pharmaceutical Sciences, Kyushu University, Fukuoka, Japan; ^4^ Departments of Pharmacology and Anesthesia, Pain Management and Perioperative Medicine, Dalhousie University, Halifax, NS, Canada

**Keywords:** inflammatory pain, neuropathic pain, microglia, primary joint afferent, ATP, arthritis

## Abstract

Increased afferent input resulting from painful injury augments the activity of central nociceptive circuits *via* both neuron-neuron and neuron-glia interactions. Microglia, resident immune cells of the central nervous system (CNS), play a crucial role in the pathogenesis of chronic pain. This study provides a framework for understanding how peripheral joint injury signals the CNS to engage spinal microglial responses. During the first week of monosodium iodoacetate (MIA)-induced knee joint injury in male rats, inflammatory and neuropathic pain were characterized by increased firing of peripheral joint afferents. This increased peripheral afferent activity was accompanied by increased Iba1 immunoreactivity within the spinal dorsal horn indicating microglial activation. Pharmacological silencing of C and A afferents with co-injections of QX-314 and bupivacaine, capsaicin, or flagellin prevented the development of mechanical allodynia and spinal microglial activity after MIA injection. Elevated levels of ATP in the cerebrospinal fluid (CSF) and increased expression of the ATP transporter vesicular nucleotide transporter (VNUT) in the ipsilateral spinal dorsal horn were also observed after MIA injections. Selective silencing of primary joint afferents subsequently inhibited ATP release into the CSF. Furthermore, increased spinal microglial reactivity, and alleviation of MIA-induced arthralgia with co-administration of QX-314 with bupivacaine were recapitulated in female rats. Our results demonstrate that early peripheral joint injury activates joint nociceptors, which triggers a central spinal microglial response. Elevation of ATP in the CSF, and spinal expression of VNUT suggest ATP signaling may modulate communication between sensory neurons and spinal microglia at 2 weeks of joint degeneration.

## Introduction

Joint pain is a debilitating and complex feature of arthritis, which affects over 300 million people worldwide (1). Osteoarthritis (OA), for example, has an etiology that includes maladaptive repair responses to trauma, metabolic dysregulation (2), and genetic predisposition (3, 4). Intermittent inflammation is a recognised aspect of OA in some patients; however, these painful flares are often poorly treated (5). Nevertheless, there remains a strong clinical demand for better chronic pain management, which is the primary concern of arthritis patients (6).

Arthritis often affects large joints, particularly the knee (7), which is highly innervated by sensory nerves in the synovium, outer meniscus, subchrondral bone and accessory ligaments (8). These joint afferents comprise of small-diameter myelinated Aδ and non-myelinated C fibres, which convey nociceptive information, and large diameter Aβ fibres responsible for encoding non-noxious sensory information (9). Joint injury triggers a cascade of inflammatory processes that sensitize these joint afferents, leading to increased nociceptive signaling from the joint to the central nervous system (10). In addition, prolonged inflammation can cause neuronal damage and apoptosis (11). Hence, both inflammatory and neuropathic components may be implicated in joint pain (12).

Converging evidence indicates that joint pain arises because of aberrant activity in both the peripheral and central nervous systems (13). Primary joint afferents have their cell bodies in the dorsal root ganglia, and enter the central nervous system (CNS) *via* the dorsal root (14). It has been shown that prolonged firing of peripheral sensory afferents in the presence of injury triggers hyperexcitability of post-synaptic neurons in the spinal dorsal horn, resulting in central sensitization and perpetuation of chronic pain states (15).

Microglia, resident immune cells of the central nervous system (16–18), are activated in the dorsal horn of the spinal cord following peripheral inflammation and neuropathy. Previous experimental studies of arthritis report increased expression of ionized calcium binding adaptor molecule 1 (Iba1), a molecular marker of microglia and microglial reactivity (19). Disrupting microglial function with pharmacological interventions (e.g., neurotoxin targeting the microglia surface marker macrophage antigen complex-1 (MAC1 saporin) (20, 21), or non-specific blocker minocycline) reduces both inflammatory and neuropathic joint pain behaviours (19, 22). Activation of microglia promotes local upregulation of pro-inflammatory cytokines including tumour necrosis factor-alpha (TNFα) (23), interleukin 1-beta (IL-1β) (20), and interleukin-6 (IL6) (24) which are known mediators of chronic pain. Although biologic-based therapies target these cytokines with neutralizing monoclonal antibodies, pain relief may be incomplete (25). These observations together point to the involvement of central immune processes in the development and maintenance of chronic joint pain.

Multiple mechanisms are known to increase microglia reactivity in chronic pain states. Notably, adenosine triphosphate (ATP), a key substrate released in the spinal cord following peripheral nerve injury (26), drives spinal microglia activation mediated by P2X4 and P2X7 receptors (27, 28). In the current study, we demonstrate the relationship between primary afferent activity and microglial responses using the monosodium iodoacetate (MIA) model of joint pain. Pain in the MIA model has a characteristic acute inflammatory phase before developing into a mixed nociceptive/neuropathic type pain as the model progresses (29, 30). We therefore first defined the timecourse of microglial reactivity after MIA injections into adult male knee joints, and the afferent inputs required to drive microglial activation. We then defined spinal microglial reactivity in response to joint injury in female animals, and demonstrated that pharmacological silencing of primary afferents alleviates mechanical allodynia in both male and female rats.

## Materials and Methods

### Animals and Ethics Statement

Adult male (200-250g) and female (151-175g) Sprague Dawley rats were purchased from Charles River Laboratories (Sherbrooke, QC, Canada) and housed at the animal facilities of the University of Calgary or Dalhousie University, Canada. Animals were under 12:12 light/dark cycle, ambient room temperature at 22°C, with access to food and water *ad libitum*. All animal experiments were approved by the University of Calgary and Dalhouse University Animal Care Committees in accordance with the guidelines of the Canadian Council on Animal Care. All tests were conducted by an experimenter blinded to the treatments.

### Intra-Articular Injections

Animals were anaesthetized with inhalation isoflurane (induction at 5%, maintenance at 2% in 100% oxygen) and prepared for sterile injections. The left knee was shaved and flexed at a 90° angle, each drug was injected into the intra-articular space using a 30-gauge needle. All drugs were dissolved in sterile saline (0.9%). To induce joint degeneration, 25 µL of 80 mg/mL monosodium iodoacetate (MIA, Sigma, I2512) was administered as previously described (20). To elucidate the contributions of C- (slow and dull nociceptive) and A- (fast and sharp nociceptive) joint primary afferents, the membrane impenetrable lidocaine sodium channel blocker QX-314 (2%, Sigma, 552233) was co-administered daily up to 3 days after MIA injections with either bupivacaine (10 µg, Sigma, B5274), capsaicin (10 µg, Sigma, M2028) or flagellin (1 µg, InvivoGen, vac-fla), targeting all sensory neurons, C- and A- afferents respectively (31–33). Responses of QX-314 coadministeration were compared to saline-treated controls.

### Joint Afferent Electrophysiology

Animals were anesthetized with urethane (2 g/kg, intraperitoneally) 1 or 7 days following MIA or saline administration and single unit recordings were carried out as previously described (34). In a supine postion, animals were artificially ventilated with 100% oxygen (Harvard Apparatus, MA, USA). An incision was made in the skin of the medial aspect of the hindlimb and the saphenous nerve was isolated. The muscle relaxant gallamine was administered (50 mg/kg, intravenously) to eliminate hindlimb neuromuscular activity, and the saphenous nerve was cut in the inguinal region to prevent spinal reflexes. Fine neurofilaments were subsequently teased and placed over a platinum recording electrode for measurement of joint afferent activity. The mechanosensitivity of Aδ and C joint afferent fibers was assessed by applying rotational force to the knee joint and counting the evoked response. The rotational force was measured by an in-series torque meter and was considered a noxious stimulus when it occurred outside the normal range-of-motion of the joint. Rotations were held for 5-seconds and applied three times over 15-minutes. The mean number of action potentials that fired in response to joint rotation were calculated offline using Spike2 software (Cambride Electronic Design, Cambridge, UK). Spontanous activity was also measured as defined by any activity of joint afferents that occurred in the absence of joint movement; the percentage of fibres from a treatment group that exhibited spontaneous firing was also recorded.

### Von Frey Tests for Measurement of Mechanical Allodynia

Mechanical allodynia was assessed by measuring the hindpaw withdrawal thresholds to von Frey filaments. Animals were placed in Plexiglas^®^ boxes on a grid surface to allow filament access to the hindpaws. Animals were habituated to the testing apparatus for 30 minutes prior to behavioural testing, before (baseline) and up to two weeks after MIA injections. von Frey hairs were applied to the central region of the plantar surface, the simplified up-down method (SUDO) was used to calculate the paw withdrawal thresholds (35).

### Dynamic Weight Bearing

The distribution of weight borne on the hindlimbs and paw contact surface area were quantified using a dynamic weight bearing system (Bioseb) (20). Animals were placed in a Plexiglas chamber and allowed to move freely. The animals were tested before (baseline) and up to two weeks after MIA injections. The floor of the chamber has a sensor array that measures the position, surface area, and level of applied pressure (weight). A camera was mounted at the top of the chamber to record the animal’s movements. The sensor data and camera footage were analyzed together to calculate the weight applied by each of the animal’s limbs and the surface area of the paws in contact with the walking surface over a 3-min recording period.

### Immunohistochemistry

Rats were anaesthetized with pentobarbital (Bimeda-MTC Animal Health Inc.) and perfused transcardially with phosphate-buffered saline (PBS) followed by 4% paraformaldehyde (PFA, Sigma, P6148). The lumbar(L)3-4 spinal cord was sectioned using a freezing cryostat at a thickness of 40 µm (36). Free-floating spinal cord sections were incubated overnight at 4°C in goat anti-Iba1 (1:500, Novus Biological, NB100-1028), followed by flurochrome-conjugated secondary antibodies donkey anti-goat Alexa 647 (1:1000, Invitrogen, A-21447) for 3 hours at room temperature. Three to six coronal sections of spinal cord were processed per animal, and three to six animals were included per treatment group. Images were obtained using a Nikon A1R multiphoton microscope and a Leica SP8 confocal microscope. The average spatial density indicating the percent area of Iba1-positive immunoreactivity, was calculated per animal using ImageJ (NIH) on both ipsilateral and contralateral spinal dorsal horns. Background, i.e., non-microglial, fluorescence was measured for control rats; once determined, all images were processed using the same threshold value in ImageJ software.

### Cerebrospinal Fluid Extraction and ATP Measurement

Cerebrospinal fluid (CSF) was obtained from anaesthetized animals (inhalation isoflurane, induction at 5%, maintenance at 2% in 100% oxygen) mounted on a stereotaxic frame (Kopf Instruments, USA). The head and neck were shaved and a midline incision was made to expose the cisternae magna, the incised area was blotted dry. A pulled glass capillary was inserted through the dura mater at a parallel angle to collect CSF *via* capillary action, samples contaminated with blood were excluded. 1 µM of ARL67156 (Sigma, A265) was added to samples to prevent ATP degradation. ATP levels were evalutated using the ATP determination kit (Life Technologies, A22066) based on firefly luciferase and its substrate D-luciferin. Using bioluminescence detected using a FilterMax F5 plate reader at 27°C, ATP concentration was calculated.

### VNUT mRNA Expression Analysis in Spinal Cord

Under terminal anaesthesia, the Lumbar(L)3-4 spinal cord was isolated and flash frozen in liquid nitrogen. The tissue was homogenized in polysome buffer and the supernatant was collected and purified using an RNeasy Micro Kit (Qiagen, 74004). Total RNA concentration was determined using NanoDrop 1000 (Thermo Fisher Scientific), and reversed-transcribed to complementary DNA (cDNA) using SuperScript™ III Reverse Transcriptase (Invitrogen, 18080093). The copy number of VNUT transcripts were determined by RT-qPCR. Briefly, VNUT primers were added to SYBR Green PCR master mix (Biorad, 1725270) in StepOne Plus RT-PCR System (Applied Biosciences). Fold enrichment was calculated using the ΔΔC_t_ method with normalization to glyceraldegyde-3-phosphate dehydrogenase (GAPDH). Primer sequences are as follows:

GAPDH rat: (forward) CCCCCAATGTATCCGTTGTG (reverse) TAGCCCAGGATGCCCTTTAGT
Slc17a9 (VNUT) rat: (forward) GCGGCAGCAGAGGACAAA (reverse) AGCAACATCCCCGTCCATAG

### Statistics

Normality tests were performed on all datasets. Data are presented as mean ± standard error of mean (SEM). All data sets were analyzed using GraphPad Prism 8 software, non-parametric tests were used where data was not normally distributed. Datapoints identified as outliers (± 2 standard deviations) by the Grubbs test were excluded from analysis. Analyses of behavioural data were performed with repeated measures two-way ANOVA with Bonferroni multiple comparisons. Electrophysiological data, time course of ATP release, and microglial reactivity after MIA were performed with two-way ANOVA or with Bonferroni multiple comparisons. Spontaneous firing of joint afferents were analyzed with Chi-square tests. Iba1 immunoreactivity, ATP concentration, and Slc179a (VNUT) mRNA expression at days 7 and 14 were compared with one-way ANOVA with Bonferroni multiple comparisons or unpaired t-tests.

## Results

### Peripheral Joint Inflammation Increases Primary Afferent Activity and Spinal Microglial Responses

**Figure 1:**
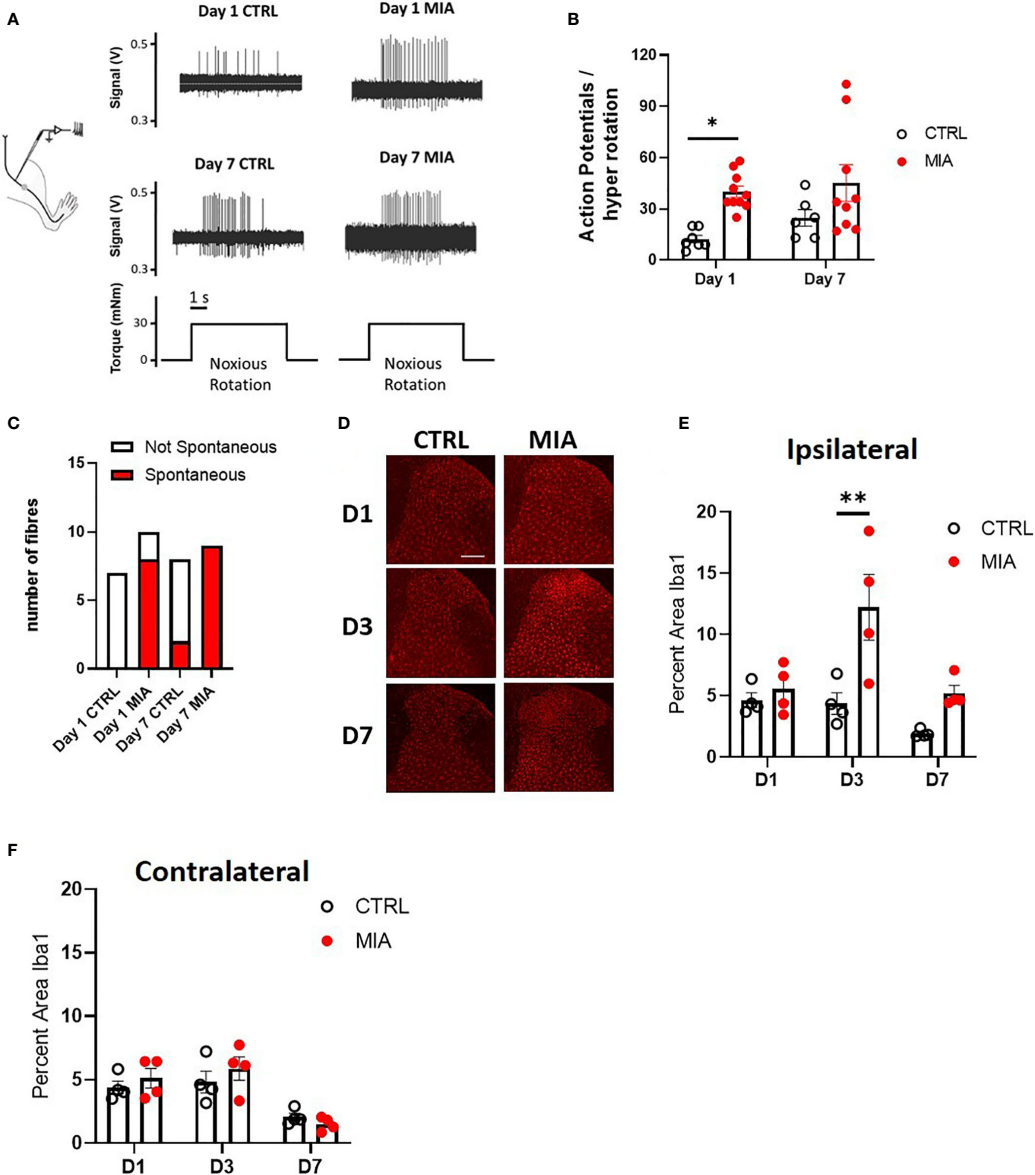
Peripheral joint inflammation increases primary afferent activity and spinal microglial response in male rats. **(A)** Schematic diagram of joint afferent recordings in primary afferents after knee injections of MIA (80mg/ml, 25μL) or saline (CTRL, 25μL). **(B)** Primary afferent activity at days 1 and 7 after MIA injections (N=6 to 9 animals per group). **(C)** Spontaneous firing at days 1 and 7 after MIA injections (N=6 to 9 animals per group). **(D)** Representative images of Iba1 immunoreactivity, a measure of microglial responses after MIA injections. Scale bar = 200μm. **(E)** Quantification of Iba1 immunoreactivity in the ipsilateral spinal dorsal horn at days 1, 3 and 7 after MIA injections (N=4 animals per group). **(F)** Quantification of Iba1 immunoreactivity in the contralateral dorsal horn at days 1, 3 and 7 after MIA injections (N=4 animals per group). *P < 0.05, **P < 0.01 comparisons between control and MIA injections.

Sensitization of joint afferent fibres was observed 1 day following the intra-articular injection of MIA in male rats (**Figure 1A**). Noxious joint rotation-evoked action potentials were increased significantly 1 day after MIA injection (CTRL vs. MIA: 12.3 ± 2.2 vs. 40 ± 3.3, p<0.05, Mixed effect analysis with Bonferroni multiple comparisons, n = 6-9 fibres, **Figure 1B**). Additionally, a large proportion (80%) of recorded fibres exhibited spontaneous activity (i.e. without noxious rotation) in the MIA cohort, while there were no appreciable spontaneously active fibres in the control animals (p<0.01, Chi-square test, **Figure 1C**). Seven days following MIA or saline administration there was no significant difference in the rotation-evoked firing rates between the two animal groups (**Figure 1B**, n = 6-9 fibres); however, all of the fibres recorded in MIA animals were spontaneously active compared to only 25% in saline control rats (p<0.01, Chi-square test, **Figure 1C**).

The timecourse of spinal microglial responses to the increased primary afferent activity was evaluated by Iba1 immunoreactivity at 1, 3, and 7 days after MIA injections (**Figure 1D**). In the ipsilateral dorsal horn, a significant increase in Iba1 immunoreactivity was detected in MIA-treated animals, two-way ANOVA analysis revealed significant effects of time F(2,18) = 7.19, P<0.01, treatment F(1,18) = 14.93, P<0.01 and interaction between time and treatment F(2,18) = 3.83, P<0.05, Bonferroni multiple comparisons showed a peak increase in Iba1 immunoreactivity at day 3 after MIA injections (control/CTRL vs. MIA, day 3: 4.36 ± 0.8 vs. 12.2 ± 2.7, P<0.01, **Figure 1E**). No appreciable changes in Iba1 immunoreactivity were detected in the contralateral dorsal horn of MIA injected animals compared to controls at any timepoints tested (**Figure 1F**).

### Early Pharmacological Silencing Of Primary Afferents Alleviates Mechanical Allodynia and Microglial Reactivity During Knee Joint Inflammation

**Figure 2:**
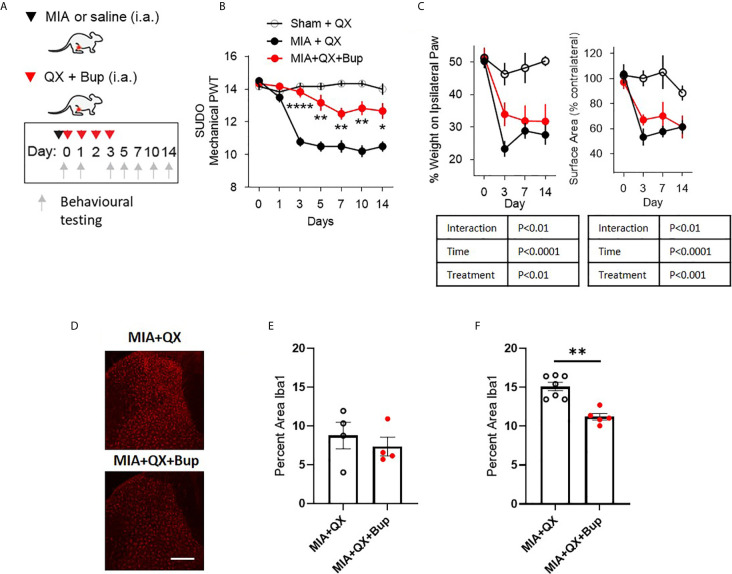
Early pharmacological silencing of primary afferent alleviates mechanical allodynia and spinal microglial response. **(A)** Schematic diagram for MIA, QX-314 (QX, 2%/25μL) and bupivacaine (Bup, 10μg/25μL) administration. **(B)** Mechanical withdrawal thresholds after QX, MIA + QX alone and MIA with QX+ Bup, animals receiving QX alone after MIA injections developed significant mechanical allodynia, which was rescued by co-administration of QX and Bup (N=6 to 7 animals per group). **(C)** Effects QX and Bup co-administration on dynamic weight bearing behaviours after MIA injections (N=6 to 7 animals per group). **(D)** Representative images of Iba1 immunoreactivity in the ipsilateral dorsal horn 7 days after MIA injections with or without Bup. Scale bar = 200μm. **(E, F)** Quantification of Iba1 immunoreactivity in the ipsilateral dorsal horn of QX and QX+Bup treated animals 7 days **(E)** and 14 days **(F)** after MIA injections (N=4 to 7 animals per group). *P < 0.05, **P < 0.01, ****P < 0.0001 comparisons between MIA+QX and MIA+QX+Bup.

To study the relationship between primary afferent activity and microglial response in the spinal dorsal horn during joint inflammation, we used a pharmacological approach to transiently silence joint afferent activity and examined the impact on pain behaviours and spinal microglial reactivity in male arthritic animals. We used QX-314, a membrane *impermeable* lidocaine derivative that must gain entry into a cell to block sodium channel conductance. First, QX-314 (QX, 2%, 25µL) was injected intra-articularly with bupivacaine (Bup, 10µg/25µL), a local anaesthetic that inhibits the activity of the nerve innervating the ipsilateral knee joint, at the time of intra-articular injection and daily for 3 days after MIA (or saline) injections (**Figure 2A**).

QX+Bup produced a long-lasting inhibition of joint pain behaviours. Compared to animals that received QX alone, QX+Bup significantly attenuated the progress of mechanical allodynia. Two-way repeated-measures ANOVA analysis revealed significant effects of time F(6,16) = 24.2, P<0.0001, treatment F(2,16) = 53.52, P<0.0001, and interaction between time and treatment F(12,96) = 12.61, P<0.00001. Bonferroni multiple comparisons revealed that this attenuation was most pronounced at day 3 following MIA injections, and was sustained for up to two weeks (**Figure 2B**).

An overall effect of QX+Bup on weight bearing deficits was detected, quantified by percentage of weight borne and contact paw surface area (**Figure 2C**). Mixed-effects analysis revealed significant effects of treatment F(2,16) = 15.31, P<0.01, time F(6,16) = 22.18, P<0.0001, and interaction between time and treatment F(6,47) = 4.161, P<0.01 on percentage weight borne on the ipsilateral paw. Mixed effects analysis revealed significant effects of treatment F(2,16) = 10.91, P<0.001, time F(6,47) = 15.17, P<0.0001, and interaction between time and treatment F(6,47) = 3.45, P<0.01 on paw contact surface area. However, Bonferroni multiple comparisons showed no difference between MIA+QX and MIA+QX+Bup groups at any individual timepoint tested.

We next assessed the effects of MIA+QX+Bup on microglial reactivity at days 7 and 14 after MIA injections (**Figure 2D**). Unpaired t-tests revealed no changes at day 7 (**Figure 2E**), but significant decrease in dorsal spinal horn Iba1 immunoreactivity at day 14 after MIA injections in MIA+QX+Bup treated animals (QX vs. MIA+QX+Bup, 15.1 ± 0.5 vs. 11.2 ± 0.4, P<0.01, **Figure 2F**).

### Selective Silencing of Primary Afferents Reveals the Role of Aβ- and C-Fibres in Mediating Pain Hypersensitivity and Microglial Reactivity

**Figure 3:**
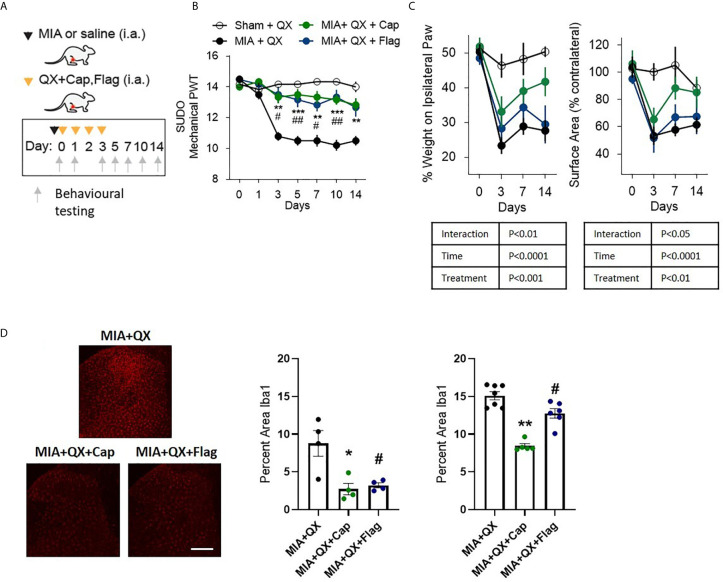
A and C primary afferent activity contributes to mechanical allodynia and spinal microglial response. **(A)** Schematic diagram for MIA, QX-314 (QX, 2%/25μL) alone, and co-administration of QX and capsaicin (QX + Cap, 10μg), and flagellin (QX + Flag, 1μg). **(B)** Mechanical withdrawal thresholds after MIA injections in QX, QX + Cap and QX + Flag (N=6 to 7 animals per group). **(C)** Effects of QX + Cap and QX + Flag on dynamic weight bearing behaviours after MIA injections (N=6 to 7 animals per group). **(D)** Representative image of Iba1 immunoreactivity in the ipsilateral dorsal horn 7 days after MIA injections in QX, QX + Cap and QX + Flag treated animals. Scale bar = 200μm. **(E, F)** Quantification of Iba1 immunoreactivity at day 7 **(E)** and day14 **(F)** after MIA injections in QX, QX + Cap and QX + Flag treated animals (N=4 to 7 animals per group). *P < 0.05, **P < 0.01, ***P < 0.001 comparisons between QX and QX + Cap, ^#^P < 0.05, ^##^P < 0.01, comparisons between QX and QX + Flag.

To pinpoint the subtype of primary afferents responsible for joint inflammatory pain and spinal microglial activity, selective inhibition with QX (2%/25µL) co-administered with either capsaicin (Cap, 10µg) or flagellin (Flag, 1µg) was used to target C- and Aβ fibres respectively in male rats. Similar to the above experiments, QX+Cap and QX+Flag were administered into the intra-articular space of the ipsilateral knee joint, at the time of MIA injections and daily for 3 consecutive dates following MIA injections (**Figure 3A**). Mechanical allodynia was assessed for 2 weeks after MIA injections. Compared to animals receiving QX alone, two-way ANOVA analysis revealed significant effects of treatment F(3,21) = 1.92, P<0.0001, time F(6,21) = 18.08, P<0.0001 and interaction between treatment and time F(18,126) = 6.382, P<0.0001. Bonferroni multiple comparisons showed significant alleviation of mechanical allodynia at days 3, 5, 7, 10 and 14 when treated with MIA+QX+Cap, and at days 3, 5, 7 and 10 days after MIA+QX+Flag (**Figure 3B**).

QX+Cap or QX+Flag also changed the overall weight-bearing responses throughout the two-week testing period. Mixed-effects analysis revealed significant effects of treatment F(3,21) = 8.845, P<0.001, time F(6,21) = 32.57, P<0.0001, and interaction between time and treatment F(9,62) = 3.116, P<0.01 on percentage weight borne in ipsilateral paw. Mixed-effects analysis also revealed significant effects of treatment F(3,21) = 6.27, P<0.01, time F(6,21) = 17.27, P<0.0001 and interaction between time and treatment F(9,62) = 2.29, P<0.05 on paw contact surface area. However, Bonferroni multiple comparisons showed no difference between QX alone compared to MIA+QX+Cap and MIA+QX+Flag at any individual timepoints tested (**Figure 3C**).

Inhibition of Aβ- or C-fibres also decreased microglial reactivity after MIA injections (**Figure 3D**). At day 7, Kruskal-Wallis analysis revealed a significant effect of treatment F(3,12) = 6.73, P<0.05, Bonferroni multiple comparisons showed significant reduction of Iba1 immunoreactivity in MIA+QX+Cap and MIA+QX+Flag treated animals compared to QX alone (MIA+QX: 8.7 ± 1.7, MIA+QX+Cap: 2.7 ± 0.8, MIA+QX+Flag: 3.2 ± 0.3, MIA+QX vs. MIA+QX+Cap and MIA+QX+Flag, P<0.05, **Figure 3E**). At day 14, Kruskal-Wallis analysis revealed a significant effect of treatment F(3,18) = 12.72, P<0.0001, Bonferroni multiple comparisons showed significant reduction of Iba1 immunoreactivity in MIA+QX+Cap and MIA+QX+Flag treated animals compared to QX alone (MIA+QX: 15.4 ± 0.5, MIA+QX+Cap: 8.4 ± 0.3, MIA+QX+Flag: 12.6 ± 0.6, QX vs. MIA+QX+Cap, P<0.01 and MIA+QX+Flag, P<0.05, **Figure 3F**).

### Joint Injury Increases Spinal Expression of ATP and the ATP Transporter VNUT at Two Weeks

**Figure 4:**
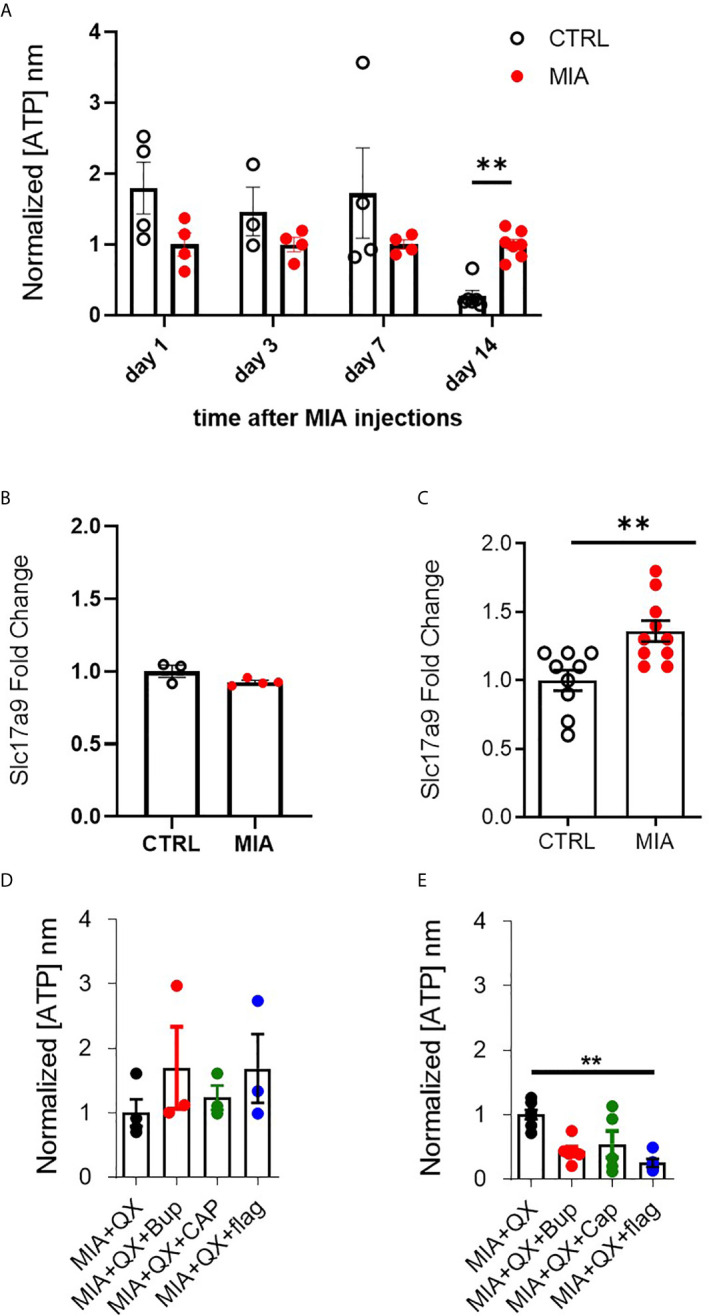
VNUT and ATP expression following MIA-induced joint injury. **(A)** Timecourse of ATP content after MIA injections in male rat cerebrospinal fluid (CSF) (N=3 to 8 animals per group). **(B)** Expression of Slc17a9 mRNA transcripts in CTRL and MIA injected animals at day 7 (N=3 CTRL, 4 MIA) and **(C)** day 14 (N=9 CTRL, 10 MIA). **(D)** ATP content in CSF 7 days after MIA injections in QX-treated animals. Kruskal-Wallis analysis detected no significant changes (N=3 to 4 animals per group). **(E)** ATP content in CSF 14 days after MIA injections in QX-treated animals (N=5 to 7 animals per group). **P<0.01.

ATP plays an important role in modulating glial activity within the central nervous system (CNS) (37–39). Cerebrospinal fluid (CSF) of male rats was collected 1, 3, 7, and 14 days after MIA injections, to evaluate ATP levels in CSF as an index of CNS neuroinflammation. Two-way ANOVA analysis detected significant effects on time F(3,28) = 4.813, P<0.01 and interaction between time and MIA treatment F(3,28) = 2.923, P<0.01, Bonferroni multiple comparisons revealed a significant increase in ATP in MIA injected animals compared to control at day 14 (CTRL vs. MIA, 0.3 ± 0.1 vs. 1 ± 0.1, P<0.01, **Figure 4A**). We also found that transcripts of VNUT (Slc179a) was unchanged at day 7 (**Figure 4B**), but upregulated in MIA injected animals at day 14 (CTRL vs. MIA, 1 ± 0.1 vs. 1.4 ± 0.1, P<0.01, **Figure 4C**).

Circulating ATP in CSF was measured 7 and 14 days after MIA and QX silencing. At day 7 in QX-treated animals, no changes in ATP levels were detected (**Figure 4D**). Significant changes were detected at day 14, Kruskal-Wallis analysis detected a significant effect of treatment, P<0.01, Dunn’s multiple comparisons revealed a significant reduction of ATP in QX + Flag treated animals compared to QX alone (MIA+QX vs. MIA+QX+Flag: 1 ± 0.1 vs. 0.3 ± 0.1, P<0.01, **Figure 4E**).

Hence, spinal ATP release is increased 2 weeks after MIA-induced joint damage, but is blunted by selective silencing of primary joint afferents.

### Peripheral Joint Injury-Induced Mechanical Allodynia is Alleviated by Pharmacological Silencing of Primary Afferents in Female Rats

**Figure 5:**
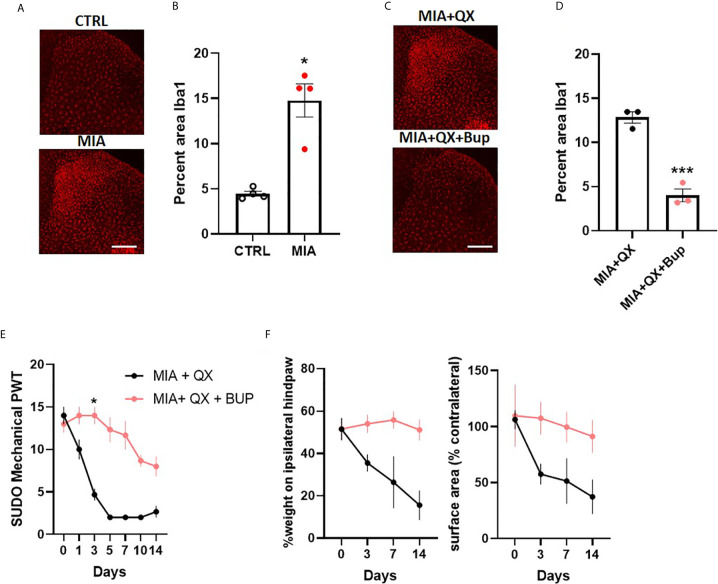
Peripheral joint inflammation induced mechanical allodynia is alleviated by pharmacological silencing of primary afferents in female rats. **(A)** Representative image of Iba1 immunoreactivity in the ipsilateral dorsal horn two weeks after MIA injections into the knee joint, and after co-administration of QX-314 (QX, 2%/25μL) and bupivacaine (Bup, 10μg/25μL). Scale bar = 200μm. **(B)** Quantification of Iba1 immunoreactivity at two weeks after MIA injections. (N=4 animals per group). **(C)** Representative image of Iba1 immunoreactivity in the ipsilateral dorsal horn at two weeks post-MIA after QX+Bup co-administration. Scale bar = 200mm. **(D)** Quantification of Iba1 immunoreactivity at two weeks after MIA and QX+Bup injections (N=3 animals per group). **(E)** Mechanical withdrawal thresholds after MIA + QX and MIA with QX+ Bup, animals receiving QX alone after MIA injections developed significant mechanical allodynia, which was rescued by co-administration of QX and Bup (N=3 animals per group). **(F)** Effects QX and Bup co-administration on dynamic weight bearing behaviours after MIA injections (N=3 animals per group). *P < 0.05, ***P < 0.001.

To investigate sex differences in joint pain mechanisms, spinal microglial reactivity was assessed at two weeks after MIA-induced joint injury (**Figure 5A**). Unpaired t-tests revealed a significant increase in the ipsilateral spinal dorsal horn Iba1 immunoreactivity in MIA animals compared to controls (CTRL vs. MIA, 4.5 ± 0.3 vs. 14.8 ± 1.8, P<0.05, **Figure 5B**). This increase was blunted by co-administration of QX+Bup (MIA+QX vs. QX+Bup, 12.8 ± 0.6 vs. 4 ± 0.7, P<0.001, **Figures 5C, D**).

Similar to males, QX+Bup alleviated mechanical allodynia for at least two weeks after MIA injections in female rats (treatment: F(1,4)=4.278, P<0.01; time: F(6,24)=33.26, P<0.001; interaction: F(6,24)=12.82, P<0.001; **Figure 5E**), Bonferroni multiple comparisons showed a peak effect on day 3. An overall treatment effect was also observed on weight bearing behaviours, as demonstrated by a significant increase in percentage weight borne on the ipsilateral limb (treatment: F(1,4)=20.03, P<0.05, **Figure 5F**), and paw contact surface area (treatment: F(1,4)=13.23, P<0.05).

## Discussion

Arthralgia is often refractory to current analgesics (6). Emerging evidence suggests that, in addition to local joint inflammation, neuroinflammatory interactions in the central nervous system (CNS) play a principal role in the development and maintenance of chronic joint pain (16, 19). Understanding the underlying mechanisms of central neuroimmune processing can lead to treatments that target more precisely the primary effectors of joint pain. The present study characterizes the anatomical and functional connections between peripheral sensory neuron activity evoked by joint inflammation and central neuroinflammatory processes.

Our findings indicate that an increase in nociceptive activity in joint primary afferents drives a corresponding upregulation of microglial reactivity in the ipsilateral spinal dorsal horn and transduction of sensation to higher order neurons in the CNS. Pharmacological silencing of primary afferents after the induction of joint injury by co-administering the membrane impermeable sodium channel blocker, QX-314, with bupivacaine to capture all nociceptive afferents, capsaicin to target C-fibres, or flagellin to target A-fibres (31, 33), also rescued pain sensitivity and spinal microglial responses. The increase in spinal microglial reactivity, alleviation of pain sensitivity and microglial response after co-treatment with QX-314 and bupivacaine were observed in both males and females, indicating that spinal microglia activation share a similar mechanism of encoding pain in both sexes. In the context of our findings we conclude that joint pain correlates with an early increase in nociceptive activity of primary sensory afferents and subsequent spinal microglial reactivity.

A variety of factors are known to drive spinal microglia reactivity, with purinergic signaling being a key neuron to glia mechanism causally implicated in chronic pain (38, 39). Notably, MIA-induced joint injury has been shown to increase P2X7 (20) and P2X4 (40, 41) receptor expression and elevate ATP levels within the CSF (20). We found that pharmacologically silencing A-fibres abrogated the increase in ATP two weeks after joint injury. The ATP may derive from various sources, including neurons, microglia, and astrocytes (42), and its release is modulated by VNUT (43, 44) which is upregulated in spinal dorsal horn neurons at 7 days after peripheral nerve injury (26). In our model, increased VNUT transcript levels was detected 2 weeks after MIA-induced joint injury. The elevated levels of ATP and VNUT coincides with mechanical allodynia, suggesting a possible role for ATP signaling in the late phases of joint pain.

A limitation of the present study is the use of MIA, a metabolite inhibitor to induce arthritis pain. MIA arthropathy does not fully recapitulate all the clinical features of OA. However, joints injected with MIA exhibit robust and reproducible nociceptive behaviour and joint pathology (19, 20). Another limitation is the focus on ATP signaling as the molecular determinant between sensory neuron and spinal microglia communication. In our study, we report an increase in ATP contained in the CSF of male rats two weeks after MIA injections, whereas joint afferent hyperexcitability and spinal microglia reactivity were detected within the first week of joint injury. It is possible that ATP may have a more pronounced effect two weeks after MIA-induced joint injury as compared with earlier time points. Other molecular signaling may also be playing a significant role in the early response to joint injury and the ensuing acute inflammation. For example, early detection of fractalkine, a chemokine which acts *via* the CX3CR1 receptor predominantly expressed on spinal microglia (45), is reported in a rat model of ankle joint inflammation (46). Mechanical hypersensitivity and spinal microglial reactivity were also blunted by administration of a fractalkine neutralizing antibody in a rat model of collagen-induced arthritis (47). In addition to fractalkines, other molecules involved in the caspase-signaling pathways could lead to maturation and production of pro-inflammatory cytokines (48, 49). Interestingly, cathepsin B-induced activation of pro-caspase-1 led to increase in interleukin-1β release by spinal microglia and development of inflammatory pain, which may be independent of ATP signaling (50). Investigation into other candidate molecules would further enhance our understanding in the communication between sensory nerves and central glia immediately after joint damage.

The efficacy of glial modulators in chronic pain management is recognized in both inflammatory and neuropathic conditions. As the resident immune cell of the CNS, microglia survey the environment and adopt a reactive phenotype in the presence of injuries and diseases (51, 52). Previous studies using the same model of MIA induced joint pain, confirmed the role of microglia by demonstrating an increase in the number of morphologically identified activated microglia and distal joint pain behaviours 7 days after MIA injections (19). Clinically, broad spectrum microglial inhibitors, such as minocycline, have also been shown to improve the progress of rheumatic diseases (53). In line with these observations, we found an increase in microglia reactivity and simultaneous development of mechanical allodynia (enhanced sensitivity to light touch), 3 days following MIA injections. We further showed that blocking primary afferent activity alleviated subsequent microglial activation and mechanical allodynia, providing the proof-of concept that primary afferent activity from the inflamed joint drives spinal microglial responses and mechanical allodynia. Current therapeutics targeting microglia lack cell-type specificity (54). Tissue resident immune cells are also under strict molecular regulations (55), and resident microglia in the CNS can repopulate as early as 5 days after successful depletion (56). A detailed understanding of mechanisms controlling microglial reactivity is therefore necessary for developing microglia-specific therapeutics that mitigate joint pain. Our findings that primary afferent activity governs spinal microglial response provides another avenue to augment neuroimmune processing relating to joint pain.

Furthermore, we observed a reduction in MIA-induced distal joint pain behaviours and microglia reactivity after pharmacological silencing of A and C-fibres. It was long established that sensitization of non-peptidergic, C- primary afferents encodes for central sensitisation and prolonged pain states (57, 58). Involvement of peptidergic, A-primary afferents has also been observed in various chronic pain conditions, whereby abnormal pain sensitivity may be generated by activation of low-threshold mechanoreceptive neurons which usually encode for innocuous sensations (59, 60). It has been shown in humans that enhanced allodynia and responses to punctate stimuli are mediated by A-fibre activity (61, 62). In rats, large diameter myelinated A-fibres are responsible for spinal microglial reactivity and proliferation immediately after peripheral nerve injury (63). Importantly, we explored the subclasses of primary afferent input in initiating spinal microglial responses, and identified differences in the timecourse of this response. We report that pharmacological silencing of both A- and C-fibres reduced microglial reactivity at 7 days and 2 weeks after MIA injections. These results, in addition to previous reports, indicate that multiple afferent inputs can trigger nociceptive and neuroimmune responses in the context of joint pain (64).

Symptoms of arthritic pain include hyperalgesia, allodynia, and spontaneous pain (65). Herein, we measured these symptoms with von Frey hair evoked allodynia, dynamic weight bearing tests assess non-evoked, spontaneous pain behaviours. In our study we observed a strong inhibition on mechanical allodynia upon primary afferent silencing, and a milder effect on weight bearing. In comparison to static weight bearing paradigms, including incapacitance tests, experimental rodent models can freely explore the testing chambers, the overall measurements encompass natural locomotive behaviours and gait (66). Of note, our pharmacological interventions target primary afferents of the nociceptive subclasses. Within the peripheral nerve bundle and the dorsal root ganglia, other subclasses of primary afferents including proprioceptors (67) would influence the regulation of posture and movement (68). Overall, our results suggest that blocking primary afferent activity attenuates joint pain related secondary allodynia. Other characteristics of spontaneous pain behaviours including guarding, scratching, or flinching may also be explored to further elucidate the impact of primary afferent blockade (69).

In summary, our findings suggest that primary afferent activity contributes to spinal microglial responses and mechanical allodynia following MIA-indued joint injury. Thus, therapies aimed at inhibiting primary joint afferent activity may mitigate pain development after joint injury.

## Data Availability Statement

The raw data supporting the conclusions of this article will be made available by the authors, without undue reservation.

## Ethics Statement

The animal study was reviewed and approved by University of Calgary and Dalhousie University Animal Care Committees, and are in accordance with the guidelines of the Canadian Council on Animal Care.

## Author Contributions

CK, YK, MM, and MO’B performed experiments and analyzed data. CK, YK, MM, JRM, and TT designed the study and conceived experiments. CK, YK, and TT wrote the manuscript. All authors contributed to the article and approved the submitted version.

## Funding

This work was supported by grants from the Vi Riddell Program for Pediatric Pain, Natural Sciences and Engineering Research Council of Canada (RGPIN06289), and the Canadian Institutes of Health Research (CIHR, PJT162271) awarded to TT. CK is supported by a CIHR Postdoctoral fellowship and MM was supported by Alberta Innovates Studentships.

## Conflict of Interest

The authors declare that the research was conducted in the absence of any commercial or financial relationships that could be construed as a potential conflict of interest.
